# Fit Accuracy of Complete Denture Base Fabricated by CAD/CAM Milling and 3D-Printing Methods

**DOI:** 10.1055/s-0042-1757211

**Published:** 2022-12-13

**Authors:** Kanyakorn Charoenphol, Chaimongkon Peampring

**Affiliations:** 1 Department of Prosthetic Dentistry, Prince of Songkla University, Hat Yai, Songkhla, Thailand

**Keywords:** denture, three-dimensional printing, computer-aided design

## Abstract

**Objective** Digital complete denture fabrication can be accomplished by either milling or three-dimensional (3D)-printing approach in which minimal distortion during processing contributes to effective denture base adaption, which leads to good denture retention. The purpose of this study was to compare the fit accuracy of milled and 3D-printed complete denture bases.

**Materials and Methods** The reference edentulous maxillary arch model was scanned to generate virtual denture bases using computer-aided manufacturing software that exports as standard tessellation language files. Denture bases were constructed using a milling and 3D-printing technique using digital light processing method ( *n* = 10). Intaglio surfaces of denture bases were scanned and superimposed on the reference model. The fit accuracy was quantified as root mean square error and evaluated statistically using independent *t* -test comparisons with a significance level of 0.05.

**Results** Milled denture bases were significantly more accurate in adaptation than 3D-printed dentures in the overall intaglio area and primary bearing area of denture bases. 3D-printed denture bases demonstrated significantly greater accuracy in adaptation than milled denture bases in the peripheral/posterior palatal seal area.

**Conclusion** Milled denture bases fit better in the overall and primary stress-bearing areas than 3D-printed dentures, while 3D-printed dentures appeared more accurate in the peripheral seal area, which had a minor undercut that is not suitable for using milling technology.

## Introduction

Edentulism can reduce the quality of life associated with dental health by affecting appearance, phonation, and function that can be restored by placing new removable dentures. 1

In complete denture fabrication, a variety of techniques had been used. 2 The purposes of each technique are to provide prosthesis with ultimate mucosal adaptability and reduce processing error resulting in good retention, support, and stability. 3 Conventional complete denture fabrication has been reliable for decades. However, the clinical protocols involved in the production of a conventional complete denture may be complicated, time-consuming, and difficult to control quality from the laboratory process. 2 4 Compression molding is the most extensively used technique and heat-polymerized polymethyl methacrylate (PMMA) is the most common material used in conventional complete denture fabrication. However, denture bases made of heat-polymerized PMMA deform during processing, resulting in linear deformation of 0.45 to 0.9%. 5

Complete denture fabrication using computer-aided design/computer-aided manufacturing (CAD/CAM) technology was first reported in the early 1990s. 6 Fewer patient visits, a simplified laboratory process resulting in less error during the denture-making process, and the ability to fabricate replacement prostheses rapidly based on stored data are all advantages of a digitally manufactured complete denture. 7 8 This is highly helpful for elderly people who have underlying diseases and having difficulty to come to dental office. 9 There are three processes in CAD/CAM workflow. Data collection and CAD are the first two steps in the process. The last step, CAM process, can be using either additive manufacturing (three-dimensional [3D] printing) or subtractive manufacturing (milling). The milling approach is a method of fabricating dentures by removing materials from prepolymerized PMMA block to form the desirable shape. Milled dentures possess has superior mechanical qualities over conventional complete dentures due to the absence of polymerized shrinkage resulting in better retention. 10 11 The residual monomer content of PMMA block was lower than that of heat-polymerized PMMA because the block was completely polymerized in a high pressure condition. 12 Milled maxillary complete dentures have been reported to be preferred by both dentists and patients. 13 The principal disadvantage of milling is a waste product, as a large portion of the blank is left unused and wasted during the process. Moreover, milling technique has certain limitations such as the contour of the restoration relies on the size of cutting tools. If the diameter of the cutting tool is bigger than the diameter of certain components, the internal fit accuracy will be compromised, or the marginal qualities will be degraded. 9

3D-printing technique using direct light processing is the most extensively used in dentistry, for example, 3D-printed casts potentially replace conventional stone casts with clinically acceptable accuracy. 14

An object has been built up layer bylayer of photopolymerizable resin using a micromirror device and ultraviolet (UV) light to solidify. A quantitative study comparison of 3D-printed denture tissue surface adaptation to the conventional approach demonstrated that there was no statistically significant difference in adaptation between the 3D-printing and conventional fabrication groups. As a result, this study concluded that the use of 3D printing to create complete dentures for try-in visits was clinically acceptable. 15 A previous study examined the *in vitro* accuracy and retention of conventional and 3D-printed dentures. They discovered that 3D-printed dentures were more accurate and better fit than conventional dentures. 16 3D-printing has benefits over milling technology, in which it can construct more complicated material geometry since it is not restricted by milling bur accessibility. 17 Moreover, material waste can be reduced and 3D-printing machine prices are lower than milling machine prices. 18 Patients' excellent levels of satisfaction with 3D-printed dentures were reported in a follow-up after 18 months of a previous clinical research. 19 However, there has been insufficient study on comparisons of accuracy of denture bases fabricated by milling versus 3D-printing technique especially when considering the peripheral/posterior palatal seal area and primary bearing area. Therefore, the objective of this research was to compare the accuracy of the overall intaglio surface, peripheral/posterior palatal seal area, and primary bearing area fabricated by milling and 3D-printing complete dentures.

## Materials and Methods

An edentulous maxillary reference model with residual ridge morphology that resembled the type A classification of the American College of Prosthodontists was created as a reference model. 20 Three metal spheres were placed on the reference model in different positions, two on the crest of the ridge over each tuberosity and one in the center of the anterior ridge served as reference points for superimposing a virtual reference model and the intaglio surface of the denture base to verify that the measurements were taken at the same position. To generate a virtual maxillary model in CAD software (3Shape Software, 3Shape Dental System, Copenhagen, Denmark), the reference model was scanned with an extraoral scanner (E4 scanner, 3Shape Dental System, Copenhagen, Denmark). An exported standard tessellation language (STL) file was created from a scanned file.

A power analysis was investigated to determine the appropriate sample size based on previous research, assuming a large effect size and type I and type II error probabilities of 0.05 and 0.95, respectively. As a result, 10 specimens were required for each group. The denture base was created from the reference CAD maxillary model by designing a virtual denture base with a thickness of 2 mm extended to the vestibule area and saving it as an STL file using the same CAD program used to create a virtual model. Twenty denture bases were constructed using two different techniques: milled denture fabrication and 3D-printed denture fabrication.

For the milled group ( *n* = 10), denture bases were constructed using CAD software (HyperDent, FOLLOW ME! Technology, Munich, Germany) and then milled with a five-axis milling machine. A prepolymerized PMMA block (Dental PMMA Monolayer, Hunan Vsmile Biotechnology Co., Ltd, Changsha, China) with a diameter of 98 mm and a height of 25 mm was milled in dry condition.

For the 3D-printed group, denture bases were constructed using a 3D-printer (Asiga Max, Asiga, Alexandra, New South Wales, Australia), a digital light processing system. The thickness of the printed layer was fixed to 100 µm, and the light source wavelength was 385 nm. Denture bases were printed with a photopolymerized resin material (Optiprint Gingiva, Dentona, Dortmund, Germany) including aliphatic urethane methacrylate, tetrahydrofurfuryl methacrylate difunctionale methacrylate, and phosphine oxide. After the printing process was completed, the denture bases were removed from the platform and cleaned twice with 99% isopropyl alcohol for 3 minutes each, followed by 30 minutes of postpolymerization using UV polymerization equipment (Asiga Flush, Asiga) according to manufacturer's recommendation.

**Fig. 1:**
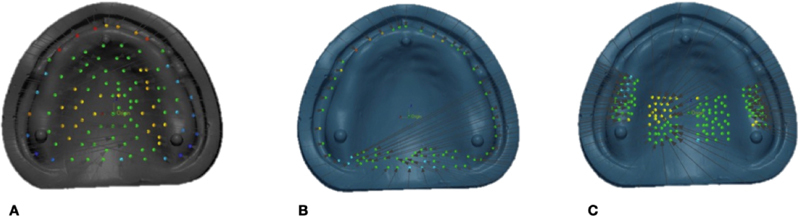
The adaptation evaluation performed in three areas: (A) overall intaglio surface with 105 measuring points, (B) peripheral/posterior palatal seal area with 72 measuring points, and (C) primary bearing area with 140 measuring points.

Using the previously mentioned extraoral scanner, the intaglio surfaces of all denture bases were scanned and saved as STL files. For accuracy measurement, each STL file of the intaglio surface of the denture was superimposed with the STL file of the reference model using first initial alignment and then best-fit alignment in a 3D measuring tool (Geomagic Control X, 3D Systems, Rockhill, South Carolina, United States). Since each point's measurements included both positive and negative values, the root mean square error (RMSE) (mm) was calculated close to zero measure accuracy. A RMSE score demonstrated the denture base as well accuracy. Accuracy was determined as the distance between the reference model's point clouds and the denture's intaglio surface. The accuracy evaluation was conducted in three locations, as indicated in Fig. 1 : (1) total intaglio surface with 105 measurement points, (2) peripheral/posterior palatal seal area with 72 measuring points, and (3) primary bearing area with 140 measuring points. Following that, a color map for qualitative expression was established. The normal variation was set to +50 µm and the critical deviation to +300 µm.

Data were statistically analyzed using SPSS 24.0 for Windows (SPSS, Chicago, Illinois, United States). The Shapiro–Wilk's test was used to verify the normal distribution and the Levene's test was used to verify the homogeneity of variance. Thus, using independent *t* -tests with a significance level of 0.05, the accuracy determined from the averages of the RMSE values was statistically compared.

## Results

The average RMSE values and standard deviations for three evaluation areas are presented in Table 1 . Independent *t* -tests demonstrated a significant difference in the total intaglio and primary stress-bearing region surface adaptation of denture bases manufactured using different methods ( *p* < 0.001) and the values indicating that milled dentures showed higher fit accuracy than 3D-printed dentures. On the contrary, 3D-printed denture bases showed lesser deviation from the reference model, implying superior adaptation, when compared with milled denture bases ( *p* = 0.09) in the peripheral/posterior palatal seal area.

**Table 1:** RMSE values (mean ± SD in mm) of three different area of measurement

Group	Mean ± SD		*p* -Value
	Milling	3D printing	
Overall surface area	0964 ± 0.0014 ^a^	1219 ± 0.0036 ^b^	<0.001
Peripheral/posterior palatal seal area	1839 ± 0.0057 ^a^	1635 ± 0.0040 ^b^	0.009
Primary bearing area	0207 ± 0.0014 ^a^	0498 ± 0.0032 ^b^	<0.001

Abbreviations: RMSE, root mean square error; SD, standard deviation; 3D, three-dimensional.

Note: Different lowercase letters in the same row indicate statistically significant difference ( *p* < 0.05).

**Fig. 2:**
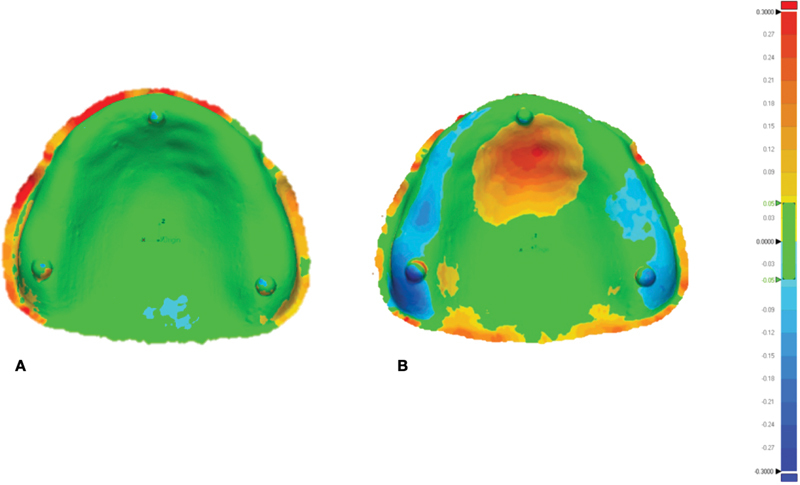
The color mapping demonstrated deviation from reference model of denture bases fabricated with different method: (A) milled denture and (B) three-dimensional-printed denture.

The color mapping of 3D measuring analysis demonstrated a positive deviation in either yellow or red, indicating that there were spaces between the denture base and the reference model. On the contrary, color mapping in cyan or blue indicated a negative deviation, indicating that the denture base was compressed relative to the reference model. The green color represents perfect intimacy between the denture base and the reference model, with an RMSE of less than 50 µm. Color mapping revealed a predominance of green color in the milled denture group, except at the periphery, and posterior palatal showed red or yellow color, whereas the 3D-printed denture groups revealed a variety of color mapping results ( Fig. 2 ).

## Discussion

Mucosal tissues covering the maxilla and mandible provide support and retention for complete dentures in edentulous jaws. Certain mucosal regions can withstand pressure, while others cannot due to the diversity of soft tissues and bone structure. 21 Generally, the primary stress-bearing area has a thick keratinized mucosa and dense cortical bone that undergoes less resorption during function. The peripheral seal is the principal factor by which the maxillary denture is retained. To maintain the peripheral seal, the periphery denture bases must maintain intimate contact with the mucosa during functions that are closely suited to the primary stress-bearing area and peripheral seal to improve support, retention, and stability. 3

Several techniques have been applied to assess the degree and location of dimension change that occurs during denture processing. These have included sophisticated two-dimensional and 3D measurements. Recently, extraoral scanners combined with surface matching software have gained popularity as a method for measuring denture base adaptation. 22 The fit accuracy or tissue adaptation of this study was obtained by RMSE by dividing the sum of all the absolute values of the deviations which are the distance between the reference model's point clouds and the surface of the scanned model. Adaptation analysis by these techniques has previously been. 23

Several studies have recently compared the accuracy of CAD/CAM milling to that of conventional methods. According to Goodacre et al, the CAD/CAM milling technique demonstrated more accuracy and repeatability than other conventional methods in the maxillary complete denture. 24 Additionally, Steinmassl et al revealed that CAD/CAM milling procedures had a higher degree of accuracy than compression molding methods. 25 The findings of this study were coincided with a study by Yoon et al. They examined the accuracy and adaptability of milled and 3D-printed mandibular denture bases and they concluded that milled bases were more accurate than printed bases. Also, the milled bases resulted in homogeneous tissue adaption in color surface mapping. However, there was no substantial difference in adaptation between milled and printed denture bases. 26 Kalberer et al examined the adaptation of milled and 3D-printed complete dentures throughout wet, dry, and wet–dry cycles. In the regions of the posterior crest, palatal vault, posterior palatal seal, tuberosity, and anterior ridge, the fit of CAD/CAM dentures was significantly better than that of 3D-printed dentures. 27 Hwang et al examined the accuracy and tissue adaptation of milled and 3D-printed maxillary bases. They reported that 3D-printed maxillary dentures demonstrated superior tissue adaption compared with milled maxillary dentures. 28 However, caution should be taken when interpreting the results, since their conclusions did not match to the color mapping result of the intaglio surface of the scanned dentures which indicated that 3D-printed maxillary dentures were more accurate than milled prostheses. Constructing complete dentures using milling or 3D printing is a relatively new trend. Although both methods require a digital 3D image file generated by CAD software, the fabrication methods are completely different. Milling denture exhibits more accuracy owing to fabrication by subtracting a PMMA block that has been industrially prepolymerized. Theoretically, the denture base should not be distorted in any dimension. 29 While 3D printing involves photosensitive liquid resins that are continuously placed onto a support structure layer by layer and polymerized by UV light, it also needs a final light-cured to ensure that complete polymerization occurs. If dentures are not completely polymerized, deformation will occur during printing. 30

The results of the study showed that there were statistically significant differences in overall intaglio, peripheral/posterior palatal seal area, and primary bearing area surface adaptation of denture bases fabricated from milling and 3D print. As a result, the null hypothesis is rejected.

The milled dentures were statistically better in fit accuracy than 3D-printed dentures except for peripheral areas where the greatest amount of milled denture mismatch was found. It is because there are some undercut in vestibular area in the referent model. Therefore, even using five-axis milling machine, the milling machine would compensatory drill to eliminate the extension of denture bases to the undercut area.

Considering some limitations of milling technique, milling units are expensive and may be appropriate for commercial centers but not for small dentistry offices. Milled dentures generated a greater quantity of material waste because of the fabrication process. Additionally, when comparing time requirements, milled dentures took ∼5 hours to produce, whereas 3D printing took ∼1.30 hours, and when comparing material costs, milled dentures were considerably more expensive than 3D printing. Both techniques were satisfactory to both the patient and the dentist. 13 19 31 Due to the dynamic movement of the mucosa in a complete denture, the tolerance of soft tissue displacement may be large. A compressive mucosal displacement of 375 to 500 µm was found in dynamic loaded maxillary denture as reported in the previous study. 32 This range of values is approximately two to three times greater than the result seen in this study. Thus, the tissue surface adaptation of the 3D-printed denture base in this study may be clinically acceptable. The limitation of this study was that the intaglio surface adaptation of denture base was determined in an extraoral condition. The oral mucosa has dynamic characteristics of compressed soft tissue which is not simulated in this study. Other factors such as saliva immersion and morphology of ridge were not considered in this study and needed to be investigated in the future.

## Conclusion

Within the limitation of this *in vitro* study, the following conclusions could be drawn. The milled dentures fit better in the overall and primary stress-bearing areas than 3D-printed dentures, while 3D-printed dentures appeared more accurate in the peripheral seal area. However, the accuracy of milled and 3D-printed denture bases in this study was a clinical acceptable level.
